# Forecasting and analyzing seasonal GHI for a SAPV system in extreme Indian climatic regions

**DOI:** 10.1038/s41598-025-23168-8

**Published:** 2025-11-12

**Authors:** Aadyasha Patel, Gnana Swathika O. V.

**Affiliations:** 1 School of Electrical Engineering, Vellore Institute of Technology, Chennai, India; 2 Centre for Smart Grid Technologies, Vellore Institute of Technology, Chennai, India

**Keywords:** GHI forecasting, Seasonal GHI, Stand-alone photovoltaic, Gaussian process regression, Affordable and clean energy, Energy science and technology, Engineering, Mathematics and computing

## Abstract

Long-term average solar radiation prediction via seasonal Global Horizontal Irradiance (GHI) forecasting is increasingly leveraging Machine Learning (ML) to uncover complex relationships in historical GHI and climatic parameters surpassing traditional methods. This study investigates seasonal GHI forecasting across four climatically distinct Indian locations namely Chennai, Jaisalmer, Leh and Mawsynram using data from the National Solar Radiation Database. Following rigorous data quality assessment and feature selection based on Spearman’s correlation and Mutual Information, eight key features are identified for training three ML models: Efficient Linear Regression (ELR), Regression Trees (RT) and Gaussian Process Regression (GPR). The GPR model demonstrates superior accuracy in seasonal GHI prediction across diverse Indian climatic conditions making it ideal for reliable and cost-efficient Stand-Alone Photovoltaic (SAPV) systems. It is observed that the GPR model achieves the lowest Root Mean Square Error (RMSE) of 0.0030, Mean Absolute Error (MAE) of 0.0022 and Coefficient of Determination (R²) of 0.9999 achieving a reduction of 189.1% in RMSE, 190.09% in MAE and 20.56% improvement in R² compared to ELR. Furthermore, the GPR model surpasses the RT model with a reduction of 124.05% in RMSE and 111.1% in MAE, together with an improvement of 0.2604% in R². While RT provides reasonably accurate forecasts, ELR shows the least reliability. These results confirm GPR’s exceptional precision and ability to capture nearly all dataset variability.

## Introduction

The prediction of long-term average solar radiation through seasonal GHI forecasting is becoming increasingly reliant on ML techniques. These approaches examine past GHI data alongside associated climatic parameters, uncovering intricate patterns and connections that conventional statistical or physical models overlook^1^. Deep learning frameworks, regression methods and time series models are trained to predict GHI over timeframes ranging from several months to a year. ML holds the promise of enhancing the precision of long-term solar resource evaluation and planning across diverse industries by analyzing historical climatic fluctuations and their influence on solar irradiance in the Indian landscape^2^.

The following literary work are relevant to this study: the research^3^ introduces an Ensemble Gaussian Process Regression (EGPR) method designed for accurate week-ahead prediction of periodic time stamped data with a specific focus on power grid generation scheduling and load demand. The EGPR method leverages the standard GPR framework but constructs an ensemble by periodically windowing the time stamped data to inform the previous covariance and mean. To address potential issues with small ensemble sizes the study implements a leave-one-out cross-validation reduction style for regularizing covariance estimations and evaluates existing reduction styles. The EGPR method was tested on a synthetic power grid dataset and Duke Energy Ohio’s load data was used to test for the real-world load data, both spanning a year of hourly measurements. The results reveal that the proposed EGPR method significantly outdoes conventional forecasting methods together with Autoregressive Integrated Moving Average, standard GPR and exponential smoothing state space model with Box-Cox transformation in predicting weekly total load demand and power generation profiles. The study highlights the effectiveness of the ensemble approach and the robust handling of covariance estimation through shrinkage techniques. The study^4^ conducted a comparative analysis of GPR, Extremely Randomized Trees, Artificial Neural Networks (ANN) and Support Vector Machines and for predicting solar PV power using yearly hourly data from a 10 MW project in the UAE. To enhance model accuracy, Bayesian Optimization (BO) and Random Search (RS) are employed for hyperparameter tuning. The results indicate that GPR consistently delivered the most accurate predictions, exhibiting the lowest error rates with both BO and RS optimization techniques. ANN ranked second in performance, reinforcing its potential for solar Photovoltaic (PV) forecasting. The study underscores the critical role of hyperparameter optimization in improving predictive accuracy.

The study^5^ evaluated Neural Prophet (NP), Facebook Prophet (FBP) and Auto-SARIMA for GHI forecasting across five-years through four districts in Rajasthan, using data from 2017 to 2019. The findings revealed that Auto-SARIMA consistently outperformed FBP and NP, achieving the lowest MAE and RMSE. Additionally, Auto-SARIMA exhibited as the best fitted model, with the lowest Bayesian Information Criterion and Akaike Information Criterion while utilizing fewer parameters. The research^6^ proposed an active learning-enhanced GPR model for both interval and point forecasting of intra-hour solar irradiance, incorporating spatial-temporal data from target and neighboring sites. The active learning process optimized input features, training data selection and GPR hyperparameters. Validation using northwest California solar irradiance data demonstrated that the suggested method outperformed up-to-date benchmarks in point forecasting exhibiting lower errors and higher R². In interval forecasting, it surpassed existing models (including persistence, autoregressive with exogenous inputs, Quantile Regression, generic GPR, bootstrap-based extreme learning machine) in forecasting reliability. The active learning-driven GPR framework significantly improves both interval and point forecasting accuracy for irradiance by effectively leveraging spatial-temporal information and optimizing model parameters, outperforming established benchmark methods.

This research^7^ investigates the effect of feature combination versus feature selection on the accuracy of ML models for GHI prediction in New Delhi. Six ML regressors namely Decision Tree (DT), Gradient Boost, Extra Tree (ET), Multiple Linear Regressor, Random Forest (RF) and Light Gradient Boost Machine were evaluated using meteorological variables. Feature selection identified five most significant predictors. When these selected features were used to train the ML models, ET regressor consistently yielded the best prediction performance with lowest Mean Absolute Percentage Error, RMSE, MAE and highest R². Comparing feature selection to feature combination, the study found that feature selection led to a notable reduction in prediction errors (8.5% for MAE, 7.72% for RMSE and 25.84% for MAPE) indicating a significant improvement in GHI prediction accuracy. The investigation^8^ proposes a novel hybrid model combining Multivariate Empirical Mode Decomposition (MEMD) by a stacked group of simpler ML algorithms (k nearest neighbor, DT regressor and ridge) for accurate and less time-complex next-hour GHI forecasting across three diverse locations in India. MEMD decomposes weather-related variables into intrinsic mode functions to address non-linearity and non-stationarity. The MEMD-stacked model significantly outperforms modern complex ML techniques like RF, ANN and Long Short-Term Memory (LSTM) achieving decrease in RMSE of 48.51% and MAE reduction of 37.52%. Seasonal analysis showed superior performance even during the unpredictable monsoon. The proposed model also demonstrates better stability and lower variance than LSTM and a stacked model without MEMD. Notably, its training time was less than the complex standalone models.

The advancement of renewable energy relies on cutting-edge analytics and ML-driven insights for a sustainable future. This paper utilizes seasonality-based feature selection to refine GHI prediction, enabling ML models to enhance SAPV system efficiency through improved forecast accuracy. This paper’s main contributions are:

- Analysis of three years’ (2017–2019) data from four climatically distinct locations across India accompanied by a thorough statistical assessment.
- Development of a systematic approach that excludes features exhibiting weak or no correlations as well as those with low or negligible MI ensuring more effective data processing.
- Proposal of refined ML models designed to generate seasonal GHI forecasts based on hourly GHI estimates.
- Assessment of model accuracy across diverse geographic regions in India to identify the most robust ML model for reliable seasonal GHI prediction.
- Compare performance of ELR and RT models with GPR to confirm the latter’s superior accuracy and near-complete data variability capture.

The paper is organized as follows: Section II outlines the data collection process and methodologies used for predicting seasonal GHI in SAPV systems. Section III presents the predicted seasonal GHI results along with comparative analysis and evaluation metrics. Finally, section IV concludes the study and discusses potential future directions in seasonal GHI forecasting.

## Data and methods

### Study area and data

India, due to its varied geography exhibits considerable climatic diversity categorized into distinct climatic zones; for instance, North India has hot, dry summers, wet monsoon and cool sometimes foggy winters while South India remains generally warm and humid with monsoon-driven wet and dry seasons with less temperature fluctuation. The Northeast mountains experience humid subtropical conditions with hot, rainy summers and mild to cold snowy winters at higher elevations contrasting sharply with the Indian desert’s extreme heat, dryness, sparse rainfall and significant diurnal temperature shifts. This study selects four distinct Indian locations namely Chennai, Jaisalmer, Leh and Mawsynram that exemplify a wide range of climatic conditions. Their classifications as outlined in Table 1 follows the Köppen climate classification system.

In this study hourly meteorological data is obtained from the National Renewable Energy Laboratory’s NSRDB Data Viewer^9,10^ by selecting the locations listed in Table 1. These locations encompass a diverse range of climatic conditions, as classified in Table 1 according to the Köppen climate classification system^11^.

**Table 1:** Köppen climate classification of selected locations in India.

Location	Region type	Köppen climate classification		Seasons		
		Letter Code	Climate type	Rainy	Summer	Winter
Chennai, Tamil Nadu	Coastal region	As	Dry summers with tropical savanna climate	July to November	March to June	December to February
Jaisalmer, Rajasthan	Hot desert region	BWh	Hot deserts	July to September	April to June	October to March
Leh, Ladakh	Cold desert region	BWk	Cold deserts	July to September	April to June	October to March
Mawsynram, Meghalaya	Wet mountain region	Cwb	Dry winters with subtropical highland climate	May to September	April	October to March

Spanning three years from January 1, 2017 to December 31, 2019 the dataset is populated in daily hourly manner that includes 15 key meteorological parameters namely, Temperature (T), Clearsky DHI (CDHI), Clearsky DNI (CDNI), Clearsky GHI (CGHI), Dew Point (DP), Diffuse Horizontal Irradiance (DHI), Direct Normal Irradiance (DNI), Ozone (O), Relative Humidity (RH), Solar Zenith Angle (SZA), Surface Albedo (SA), Pressure (P), Precipitable Water (PW), Wind Direction (WD) and Wind Speed (WS) as predictors along with one response variable, GHI, all of which are essential for evaluating SAPV power generation potential.

Table 2 summarizes the descriptive statistics of the curated dataset for the selected Indian locations offering insights into the variability and distribution of meteorological parameters essential for feature selection and model training. It includes the minimum and maximum values, standard deviation (SD), quantiles and the count for each meteorological parameter. A comprehensive analysis is performed by computing summaries of all 16 features ensuring effective feature selection. This dataset serves as the foundation for the prediction model guiding both feature selection and ML model deployment. These are the key processes for achieving accurate seasonal GHI predictions across diverse climatic regions.

**Table 2:** Descriptive statistics of meteorological parameters for four selected locations.

Descriptive statistics of Chennai								Descriptive statistics of Jaisalmer							
Variable	Min	25%	50%	75%	Max	SD	Count	Variable	Min	25%	50%	75%	Max	SD	Count
T	21.90	27.80	29.70	32.20	38.70	2.86	13,060	T	5.30	25.80	32.40	37.00	46.70	7.55	12,962
CDHI	1.00	111.00	158.00	210.00	489.00	76.61	13,060	CDHI	1.00	122.00	193.00	277.00	612.00	112.84	12,962
CDNI	0.00	415.00	624.00	741.00	955.00	237.09	13,060	CDNI	0.00	286.00	502.00	656.00	1004.00	242.14	12,962
CGHI	1.00	274.00	621.00	830.00	1045.00	306.58	13,060	CGHI	1.00	253.00	549.00	763.00	1051.00	289.95	12,962
DP	13.20	21.50	23.10	24.50	27.60	2.25	13,060	DP	−21.00	1.10	9.40	20.00	27.40	10.65	12,962
DHI	1.00	108.00	214.00	352.00	517.00	143.28	13,060	DHI	1.00	115.00	194.00	288.00	612.00	120.36	12,962
DNI	0.00	55.00	241.00	403.25	955.00	240.51	13,060	DNI	0.00	188.25	434.00	628.75	1004.00	260.07	12,962
GHI	1.00	154.00	422.00	652.00	1045.00	284.91	13,060	GHI	1.00	216.00	502.00	731.75	1051.00	293.17	12,962
O	0.22	0.25	0.26	0.27	0.29	0.01	13,060	O	0.21	0.26	0.27	0.28	0.35	0.01	12,962
RH	32.00	56.40	66.54	76.83	95.54	12.63	13,060	RH	3.18	16.18	27.21	42.56	95.35	18.46	12,962
SZA	1.69	31.92	47.17	69.00	88.99	22.82	13,060	SZA	4.33	37.97	53.46	69.84	88.99	21.24	12,962
SA	0.13	0.14	0.15	0.15	0.18	0.00	13,060	SA	0.24	0.28	0.28	0.29	0.30	0.01	12,962
P	997.00	1004.00	1007.00	1010.00	1019	4.09	13,060	P	965.00	975.00	981.00	987.00	999.00	7.23	12,962
PW	1.10	3.30	4.70	5.70	7.10	1.44	13,060	PW	0.30	1.20	2.20	3.70	7.20	1.69	12,962
WD	0.00	69.00	154.00	237.00	358.00	90.28	13,060	WD	1.00	121.00	200.00	226.00	359.00	82.68	12,962
WS	0.30	2.70	3.70	4.50	9.90	1.28	13,060	WS	0.20	1.80	2.80	4.10	11.90	1.77	12,962

Descriptive statistics of Leh								Descriptive statistics of Mawsynram							
Variable	Min	25%	50%	75%	Max	SD	Count	Variable	Min	25%	50%	75%	Max	SD	Count
T	−32.60	−3.10	6.40	13.80	27.40	10.78	13,110	T	6.60	19.80	22.80	24.60	29.50	3.64	13,062
CDHI	3.00	59.00	84.00	112.00	365.00	46.44	13,110	CDHI	2.00	85.00	122.00	191.00	531.00	82.94	13,062
CDNI	0.00	700.00	892.00	978.00	1162.00	246.15	13,110	CDNI	0.00	442.00	648.00	790.00	991.00	244.25	13,062
CGHI	3.00	286.00	585.00	828.00	1131.00	314.80	13,110	CGHI	2.00	277.00	578.00	778.00	1017.00	294.95	13,062
DP	−40.9	−16.20	−9.90	−3.80	7.60	8.51	13,110	DP	0.30	13.90	20.30	23.20	26.40	5.40	13,062
DHI	1.00	58.00	98.00	217.00	719.00	158.73	13,110	DHI	1.00	58.00	123.00	239.00	524.00	130.63	13,062
DNI	0.00	113.00	445.00	903.00	1162.00	383.28	13,110	DNI	0.00	0.00	91.50	513.00	991.00	310.04	13,062
GHI	1.00	163.00	442.00	714.00	1131.00	313.01	13,110	GHI	1.00	72.00	264.00	518.00	1010.00	262.15	13,062
O	0.22	0.25	0.26	0.28	0.44	0.02	13,110	O	0.21	0.25	0.26	0.27	0.34	0.015	13,062
RH	6.07	25.18	34.33	44.40	68.01	12.61	13,110	RH	29.76	71.18	86.32	96.92	100.00	16.16	13,062
SZA	11.68	41.67	57.85	72.69	88.99	20.07	13,110	SZA	5.72	36.57	52.44	70.37	88.99	21.66	13,062
SA	0.17	0.21	0.22	0.24	0.87	0.21	13,110	SA	0.09	0.11	0.12	0.13	0.15	0.014	13,062
P	635.00	648.00	650.00	652.00	658.00	3.75	13,110	P	885.00	897.00	902.00	905.00	913.00	4.75	13,062
PW	0.00	0.30	0.50	0.90	2.50	0.49	13,110	PW	0.40	1.70	3.10	4.80	6.20	1.59	13,062
WD	2.00	210.00	246.00	272.00	356.00	58.71	13,110	WD	0.00	165.00	202.00	227.00	360.00	59.74	13,062
WS	0.20	1.70	2.60	3.70	11.40	1.41	13,110	WS	0.30	1.20	1.70	2.40	6.50	0.89	13,062

## Methods

### Methodology

Predicting seasonal GHI is essential for SAPV power estimation, as it determines the solar radiation available for PV panel absorption. Additionally, seasonal GHI prediction plays a crucial role in optimizing PV system planning by estimating solar energy availability for each season. By leveraging meteorological data and climate trends ML models enhance forecasting accuracy, improving solar energy utilization across diverse climatic regions.

This study employs temporal validation within climatically diverse regions to assess seasonal GHI forecasting performance. The validation approach uses temporal splitting where data from 2017 to 2018 serves as the training set and 2019 data serves as the testing set, allowing evaluation of model performance on unseen time periods within the same geographic locations. The four selected locations represent extreme diversity in Indian climatic conditions according to Köppen classification, providing comprehensive coverage as presented in Table 1. This temporal generalization approach is particularly relevant for seasonal forecasting applications where models must predict GHI patterns for future time periods based on historical climatic patterns.

**Fig. 1:**
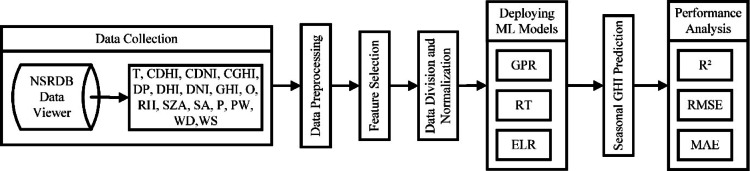
Proposed system methodology.

Figure 1. illustrates the proposed system methodology flowchart detailing the process of predicting and evaluating seasonal GHI for a SAPV system across India’s extreme climatic zones using ML models. The process begins with data collection via the NSRDB data viewer followed by data pre-processing to remove anomalous values and outliers. Next, feature selection is conducted to identify the most relevant parameters for seasonal GHI forecast using ML models. The data then undergoes division and normalization before the deployment of ML models such as ELR, RT, and GPR for prediction of seasonal GHI. The final stage is performance analysis via RMSE, MAE and R^2^ metrics which assesses the accuracy and effectiveness of these models in forecasting seasonal GHI.

### Data preprocessing

Data pre-processing ensures integrity and relevance by handling missing values and outliers to improve data quality. Additionally, data recorded outside the sunrise-to-sunset window is excluded as it holds no significance for seasonal GHI prediction thereby enhancing the accuracy of predictive models.

### Contribution of features

Feature selection plays a crucial role especially when handling high-dimensional datasets. Its primary objective is to identify and retain the most relevant features while eliminating redundant or irrelevant ones, thereby enhancing model accuracy, reducing computational complexity and minimizing the risk of overfitting. Two techniques are used for feature selection: Spearman’s rank correlation coefficient ($\:{\rho\:}_{S}$) to evaluate monotonic relationships and Normalized Mutual Information (NMI) to quantify for the dependency strength while accounting its variable scale.

Correlation analysis is a widely adopted technique for feature selection as it examines relationships between variables and helps detect dependencies or multicollinearity. Given the abundance of meteorological variables not all are equally significant for forecasting seasonal GHI. Thus, correlation analysis is essential in determining the relationship between seasonal GHI and each meteorological parameter. $\:{\rho\:}_{S}$ is employed as a quantitative measure to assess these associations^12^ as in (1). It assesses the direction and strength of monotonic relationships among features relying on ranked data rather than raw values making it robust against outliers and effective in identifying nonlinear trends.

1 $$\:{\rho\:}_{S}=1-\frac{6\sum\:{d}_{i}^{2}}{n({n}^{2}-1)}$$

$\:{d}_{i}$ rank difference per observation,

*n* total sample size.

MI^13^ measures the dependency between two variables by evaluating how much information one provides about the other. Rooted in information theory, MI is especially valuable for feature selection when dealing with nonlinear relationships. NMI refines MI by adjusting for variable scale, yielding a score between 0 and 1 as in (2) and (3). This normalization makes NMI particularly effective for comparing feature importance across datasets or different measurement scales.

2 $$\:NMI\left(X;Y\right)=\frac{MI(X;Y)}{\sqrt{H\left(X\right)\cdot\:H\left(Y\right)}}$$

3 $$\:MI\left(X;Y\right)=\sum\:_{x\in\:X}\sum\:_{y\in\:Y}p\left(x,y\right)log\left(\frac{p(x,y)}{p\left(x\right)p\left(y\right)}\right)\:$$

H(X) entropy of variable X.

H(Y) entropy of variable Y.

p(x, y) joint probability distribution of X and Y.

p(x) marginal distributions of X.

p(y) marginal distributions of Y.

X meteorological variables.

Y GHI.

Notably, some of the most significant predictors such as CGHI, CDHI and CDNI are deterministically derived from solar geometry, atmospheric models and time-location inputs. These parameters exist independently of measured irradiance values and remain available in real forecasting scenarios. Their inclusion enhances predictive performance while ensuring that the framework applies to future oriented seasonal GHI forecasting rather than being restricted to historical data fitting.

### Efficient linear regression (ELR)

ELR is an improved version of traditional linear regression designed to enhance performance when working with large datasets while minimizing computational complexity. It focuses on accurately capturing the relationship between independent and dependent variables while mitigating challenges like multicollinearity and overfitting. To improve efficiency techniques such as feature selection and regularization are commonly applied.

### Regression trees (RT)

RT is a decision tree-based approach for predicting continuous numerical values. It functions by systematically forming smaller datasets based on the values of specific features and establishing a structured hierarchy. Each division is optimized to minimize prediction errors ensuring effective segmentation of the data. RT excels at capturing non-linear relationships and managing complex datasets with variable interactions. While they are easy to interpret, they are susceptible to overfitting.

### Gaussian process regression (GPR)

GPR is a non-parametric technique that represents data as a distribution over functions, making it highly effective in modeling uncertainty in predictions. It establishes a prior distribution on functions and refines it based on observed data to generate a posterior distribution. GPR is well-suited for small to medium-sized datasets with intricate patterns, providing flexible and robust predictive capabilities.

Table 3 presents the three ML models along with their corresponding optimized hyperparameters. BO^14^ is employed to fine-tune these hyperparameters enhancing predictive performance to its optimal level. After the training phase the models undergo validation through 5-fold cross-validation. This methodology employs a five-fold cross-validation strategy involving iterative training on four folds and evaluation on the single held-out fold. This approach helps reduce the risk of overfitting and ensures a thorough assessment of model performance. The accuracy and consistency of seasonal GHI forecasting in SAPV systems are enhanced by the model’s generalizability and reliability in performance.

**Table 3:** ML models and their corresponding optimized hyperparameters.

Model	Hyperparameters	Optimized hyperparameters
ELR	Learner	Least squares
	Regularization	Ridge
	Regularization strength (λ)	0.09694
RT	Minimum leaf size	3
	Surrogate decision splits	Off
GPR	Basis function	Linear
	Kernel function	Isotropic Matern 5/2
	Kernel scale	5.9895
	Sigma	0.00010961
	Standardize data	No

#### Performance analysis

Assessing the operation of ML models is essential to warrant accuracy and reliability. Common error metrics^15^ comprise RMSE, MAE, and R². RMSE determines the square root of the mean squared differences between predicted and actual values giving greater weight to larger errors as in (4). It is susceptible to outliers and suitable when penalizing large deviations. MAE calculates the average magnitude of prediction errors without contemplating their direction as in (5). It is easy to interpret and ideal when all errors carry equal weight. R² evaluates how well the model describes the changeability of the target variable, as in (6), with values ranging from 0 to 1 where higher value indicate better performance. By utilizing these metrics collectively investigators can thoroughly assess and compare ML models to identify the most precise and consistent model for deployment.

4 $$\:RMSE=\sqrt{\frac{1}{n}{\sum\:}_{i=1}^{n}{\left({Y}_{i}-\widehat{Y}\right)}^{2}}$$

5 $$\:MAE=\frac{1}{n}{\sum\:}_{i=1}^{n}\left|{Y}_{i}-\widehat{Y}\right|$$

6 $$\:{R}^{2}=1-\frac{\sum\:{\left({Y}_{i}-\widehat{Y}\right)}^{2}}{\sum\:{\left({Y}_{i}-\stackrel{-}{Y}\right)}^{2}}$$

$$\:{Y}_{i}$$ actual values.

$$\:\widehat{Y}$$ forecasted values.

$\:\stackrel{-}{Y}$ mean of the actual values.

#### Resources utilized

Statistical analyses were performed using JMP Student Edition 18, while MATLAB R2023b was employed for the implementation of machine learning models. All computations were executed on a system equipped with an AMD Ryzen 3 7320U processor, 8 GB DDR4 memory and Windows 11 Pro operating system.

## Results and discussion

### Climate overview of the cities

**Fig. 2:**
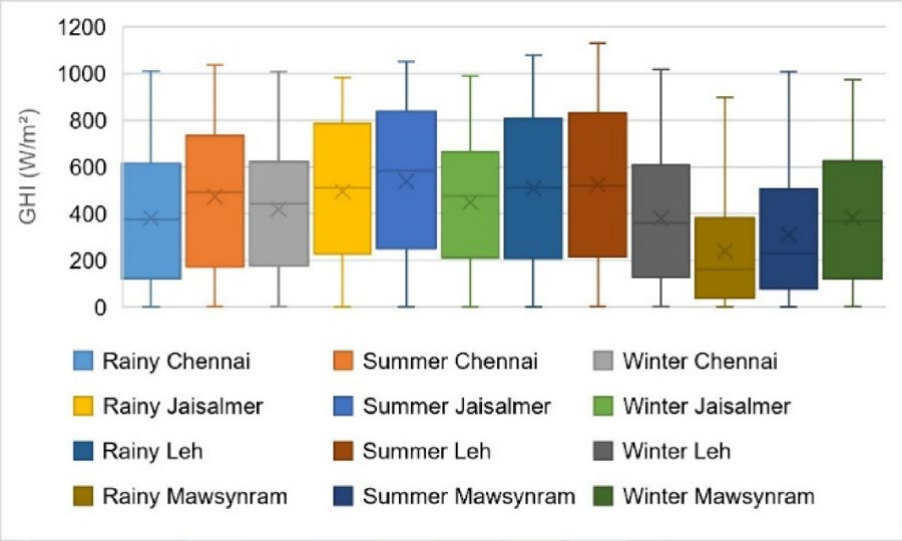
Seasonal distribution of GHI across four locations.

Figure 2. illustrates the seasonal GHI distribution across four locations over three different seasons from 2017 to 2019. The symmetrical positioning of the median line within the boxplots suggests an approximate normal distribution which is a key aspect of statistical analysis. The variations in GHI across different seasons and regions in India are evident where higher values signify greater availability of solar radiation essential for SAPV applications.

Key observations from Fig. 2. include: Mawsynram, a heavy rainfall region, exhibits high variability in GHI during the rainy season. Despite frequent cloud cover there are still periods of significant solar radiation. However, the variability highlights the impact of cloud cover on solar energy availability. Leh, a high-altitude desert region, consistently shows elevated GHI values across all seasons attributed to clear skies and reduced atmospheric absorption. Summer presents high median and mean values indicating strong solar potential while winter sees lower GHI due to shorter days and possible atmospheric attenuation. Jaisalmer, a hot desert region, maintains relatively high GHI throughout the year, making it a stable solar resource for a SAPV setup. Chennai, a coastal city, records moderate GHI values. Summer GHI is comparatively higher due to longer daylight hours and clearer skies, although humidity and cloud cover in coastal areas can reduce solar radiation levels.

These insights provide a comprehensive understanding of seasonal and regional variations in GHI, aiding efficient planning for SAPV applications.

### Influence of significant predictors

𝜌𝑆 is computed for each season and location by analyzing the relationship between 15 meteorological parameters and GHI along with their corresponding p-values. The results are summarized in Table 4. Examining the Rainy Chennai column in Table 4, it is seen that CGHI, DHI and DNI exhibit a strong positive monotonic correlation with GHI whereas SZA demonstrates a strong negative monotonic correlation with GHI. T, CDHI and CDNI show a moderate positive monotonic correlation with GHI while RH exhibits a moderate negative monotonic correlation with GHI. Weak positive monotonic correlations are displayed by O, SA, P and WD while DP, PW and WS have weak negative correlations with GHI.

In the next stage of analysis, only features exhibiting strong or moderate correlations with GHI are retained, while those with weak or no correlation are omitted. Yet, correlation alone does not definitively establish feature significance. To further improve the feature selection process and ensure statistical validity, a hypothesis test is performed to assess whether the observed correlations are genuinely meaningful or merely coincidental. The hypothesis test for feature correlation is performed using the ensuing hypotheses:

- Null Hypothesis (H0): If the p-value is greater than α, no correlation is detected.
- Alternate Hypothesis (H1): If the p-value is less than or equal to α, a non-zero correlation is present.

In this study, a Confidence Interval (CI) of 95% is established with significance level, α of 0.05. The goal is to reject H0 when the p-value is below this threshold, signifying a statistically significant relationship. To verify statistical significance p-values are calculated for all coefficients as presented in Table 4. Eight key features, highlighted in bolded italics, are identified for further analysis: T, CDHI, CDNI, CGHI, DHI, DNI, RH and SZA.

**Table 4:** Spearman’s correlation between meteorological parameters and GHI with corresponding p-values for a 95% CI.

Features	Rainy				Summer				Winter			
	Chennai	Jaisalmer	Leh	Mawsynram	Chennai	Jaisalmer	Leh	Mawsynram	Chennai	Jaisalmer	Leh	Mawsynram
***T***	0.4814	0.5464	0.5497	0.6374	0.6226	0.5437	0.4320	0.5901	0.7172	0.5492	0.3675	0.4352
	< 0.0001	< 0.0001	< 0.0001	< 0.0001	< 0.0001	< 0.0001	< 0.0001	< 0.0001	< 0.0001	< 0.0001	< 0.0001	< 0.0001
***CDHI***	0.6161	0.6963	0.6046	0.5476	0.7518	0.7989	0.5342	0.3795	0.653	0.7357	0.5350	0.4966
	< 0.0001	< 0.0001	< 0.0001	< 0.0001	< 0.0001	< 0.0001	< 0.0001	< 0.0001	< 0.0001	< 0.0001	< 0.0001	< 0.0001
***CDNI***	0.6825	0.7843	0.8147	0.5724	0.8071	0.8583	0.8279	0.5919	0.8222	0.8126	0.7842	0.6699
	< 0.0001	< 0.0001	< 0.0001	< 0.0001	< 0.0001	< 0.0001	< 0.0001	< 0.0001	< 0.0001	< 0.0001	< 0.0001	< 0.0001
***CGHI***	0.8209	0.9312	0.8742	0.6907	0.9377	0.9758	0.8891	0.5707	0.9041	0.9698	0.8720	0.7735
	< 0.0001	< 0.0001	< 0.0001	< 0.0001	< 0.0001	< 0.0001	< 0.0001	< 0.0001	< 0.0001	< 0.0001	< 0.0001	< 0.0001
DP	−0.2413	−0.14	−0.1914	0.2601	−0.3604	−0.1107	−0.1978	−0.2116	−0.2805	−0.0063	0.0470	−0.1934
	< 0.0001	< 0.0001	< 0.0001	< 0.0001	< 0.0001	< 0.0001	< 0.0001	< 0.0001	< 0.0001	0.6927	0.0034	< 0.0001
***DHI***	0.8474	0.7376	0.4886	0.8488	0.7202	0.8055	0.5907	0.8917	0.7108	0.7394	0.5213	0.6417
	< 0.0001	< 0.0001	< 0.0001	< 0.0001	< 0.0001	< 0.0001	< 0.0001	< 0.0001	< 0.0001	< 0.0001	< 0.0001	< 0.0001
***DNI***	0.8149	0.8463	0.7110	0.9333	0.8305	0.8960	0.6597	0.8502	0.7741	0.8335	0.7809	0.8121
	< 0.0001	< 0.0001	< 0.0001	< 0.0001	< 0.0001	< 0.0001	< 0.0001	< 0.0001	< 0.0001	< 0.0001	< 0.0001	< 0.0001
O	0.0672	0.081	0.0257	0.0593	−0.0604	0.0033	−0.0230	0.0125	0.1834	0.0903	−0.0231	0.0433
	< 0.0001	< 0.0001	< 0.0001	0.0002	0.0008	0.871	0.256	0.7357	< 0.0001	< 0.0001	0.1505	0.0063
***RH***	−0.4489	−0.4206	0.2062	−0.6965	−0.652	−0.3002	−0.6077	−0.5587	−0.6462	−0.4845	−0.3727	−0.6834
	< 0.0001	< 0.0001	< 0.0001	< 0.0001	< 0.0001	< 0.0001	< 0.0001	< 0.0001	< 0.0001	< 0.0001	< 0.0001	< 0.0001
***SZA***	−0.8202	−0.9139	−0.8596	−0.6903	−0.925	−0.9612	−0.8721	−0.5407	−0.8916	−0.9592	−0.8618	−0.7435
	< 0.0001	< 0.0001	< 0.0001	< 0.0001	< 0.0001	< 0.0001	< 0.0001	< 0.0001	< 0.0001	< 0.0001	< 0.0001	< 0.0001
SA	0.0478	0.041	−0.0400	−0.0398	−0.0293	−0.0308	0.0091	0.0272	0.1039	0.0921	−0.1070	−0.0154
	0.0042	0.0476	0.0496	0.0117	0.1049	0.1345	0.652	0.4617	< 0.0001	< 0.0001	< 0.0001	0.3306
P	0.0153	0.0416	−0.0418	0.1346	0.1165	0.0704	0.1286	0.2637	−0.0546	−0.1204	0.2166	0.2006
	0.3597	0.0448	0.0398	< 0.0001	< 0.0001	0.0006	< 0.0001	< 0.0001	0.0135	< 0.0001	< 0.0001	< 0.0001
PW	−0.1971	−0.0938	−0.1517	−0.1586	−0.1986	−0.0609	−0.1857	−0.3247	−0.1926	0.0019	−0.0759	−0.2203
	< 0.0001	< 0.0001	< 0.0001	< 0.0001	< 0.0001	0.0031	< 0.0001	< 0.0001	< 0.0001	0.9048	< 0.0001	< 0.0001
WD	0.0668	−0.1243	0.1419	−0.0696	−0.1629	−0.0153	0.1116	0.0735	0.0689	0.1607	0.2122	−0.0816
	< 0.0001	< 0.0001	< 0.0001	< 0.0001	< 0.0001	0.4575	< 0.0001	0.0469	0.0018	< 0.0001	< 0.0001	< 0.0001
WS	−0.0519	−0.1107	0.4419	0.0336	−0.1893	0.1425	0.3350	−0.0039	0.0618	0.1231	0.3432	0.1877
	0.0019	< 0.0001	< 0.0001	0.0333	< 0.0001	< 0.0001	< 0.0001	0.9151	0.0051	< 0.0001	< 0.0001	< 0.0001

**Fig. 3:**
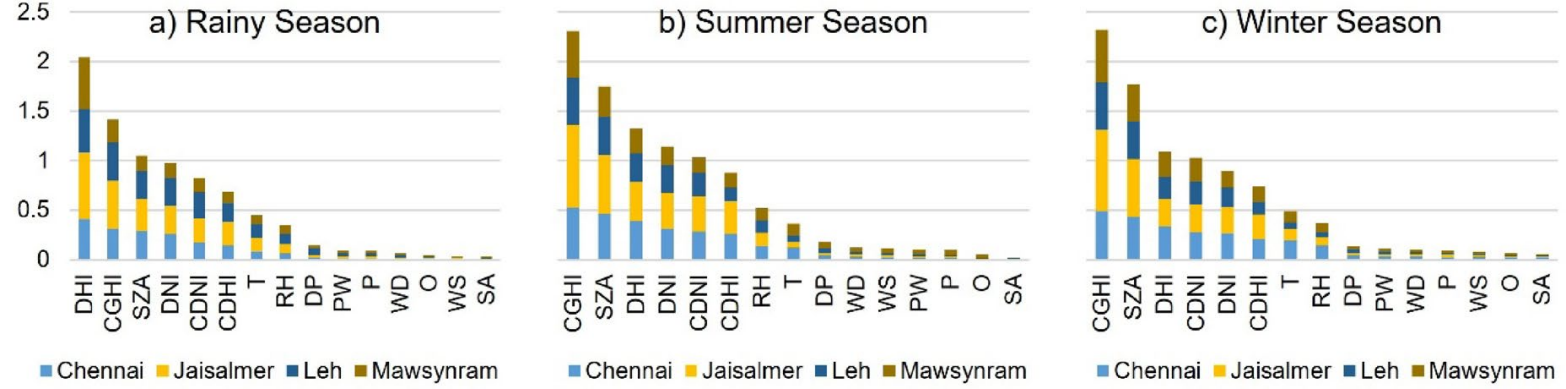
Seasonal variation of NMI relative to GHI across four locations.

To further validate these findings, the NMI of seasonal data is used to examine the relationship between meteorological features and GHI across all locations categorized by season as illustrated in Fig. 3. It consists of three stacked bar plots each corresponding to a distinct season in India. Specifically, Fig. 3a, b and c represent the rainy, summer and winter seasons respectively. Each bar within the plots denotes a specific meteorological parameter with segments color-coded to indicate contributions from all four locations. The y-axis represents the magnitude of these parameters. The chart presents the importance of features in ascending order ranging from the most significant to the least significant. The results reveal that the top eight features exhibit a strong degree of MI with GHI emphasizing their critical role in predictive modelling.

These findings reinforce the conclusions drawn from previous correlation analysis demonstrating the consistency and reliability of the feature selection process. By retaining only the most relevant features, SAPV system performance is enhanced leading to improved accuracy in seasonal GHI forecasting.

### Data division and normalization

To enhance model performance the dataset is carefully split into training and testing sets. Observations from 2017 to 2018 are designated for training, while data from 2019 is reserved for evaluation and testing. This temporal division ensures the model is trained on historical data and validated on unseen data, enabling an accurate assessment of its generalizability.

Table 2 summarizes the minimum and maximum values of various meteorological parameters emphasizing the differences in scale among the features. To address this discrepancy and eliminate bias in predictions due to differences in feature magnitudes, all variables undergo min-max normalization^16–18^ as in (7). This process adjusts each feature to a standardized range between 0 and 1 ensuring equal contribution to the model’s learning process regardless of their initial units or scales. After prediction the results are denormalized using the inverse of the normalization equation to revert them to their original scale for better interpretability. This process is crucial for ensuring consistency and accuracy in model performance especially in ML algorithms that are highly sensitive to feature scaling.

7 $$\:{X}_{norm}=\left(X-{X}_{min}\right)/\left({X}_{max}-{X}_{min}\right)$$

*X* actual value of the input variable.

$\:{X}_{norm}$ normalized value of the input variable.

$\:{X}_{min}$ minimum value of the input variable.

$\:{X}_{max}$ maximum value of the input variable.

Although the present study employs a temporal split utilizing years 2017–2018 for training and 2019 for testing in order to assess model performance over time, further validation across locations strengthens the robustness of seasonal GHI forecasting. A cross-location evaluation, where the model trains on three sites and tests on the fourth, represents an important extension that demonstrates generalizability across India’s diverse climatic regions. This direction forms part of the future scope of the work.

### Performance comparison of various predictive models

Table 5 presents a detailed performance comparison of ML models across various Indian locations, each exhibiting distinct climatic characteristics. The ELR, RT and GPR models follow a structured training, validation and testing process for each seasonal dataset enabling thorough evaluation across different temporal and climatic conditions. The predictive performance of each model is assessed and benchmarked against key evaluation metrics for every season, offering an in-depth analysis of model effectiveness in handling seasonal variations in solar irradiance and meteorological parameters. Highlighting the outstanding results, the most favorable predictions are emphasized in bold for clarity.

The GPR model demonstrates superior performance across all three evaluation metrics for predicting seasonal GHI. It achieves the lowest RMSE of 0.0030 which is 189.1% and 124.05% lower than the ELR and RT models respectively. Additionally, GPR exhibits the smallest MAE of 0.0022 reflecting 190.09% and 111.1% lower prediction errors compared to ELR and RT models respectively. Furthermore, the R² for GPR is remarkably high at 0.9999 surpassing ELR and RT by 20.56% and 0.2604% respectively. This comparative analysis highlights the GPR model’s exceptional accuracy in predicting seasonal GHI and its ability to account for nearly all variability within the dataset.

The RT model exhibits strong performance with a relatively low RMSE of 0.0128 and a MAE of 0.0077 complemented by a high R² of 0.9973. These metrics indicate that the RT model provides a reliable fit for the dataset. In contrast, the ELR model records the highest RMSE of 0.1070, the largest MAE of 0.0864 and the lowest R² value of 0.8135 among the three models. This suggests that ELR is the least accurate in predicting seasonal GHI for Chennai during the rainy season compared to RT and GPR.

An in-depth analysis of Table 5 reveals a clear pattern indicating that GPR model consistently delivers superior performance across all seasons and regions. However, an exception is observed in the rainy and winter seasons of Mawsynram where the RT model demonstrates a slight advantage over GPR. In the rainy season of Mawsynram the RT model outperforms the GPR model achieving a 29.51% lower RMSE and a 53.42% lower MAE. Additionally, the RT model records a R² that is 0.1504% higher than GPR. Similarly, in the winter season of Mawsynram the RT model demonstrates superior accuracy with RMSE and MAE values that are 5.243% and 60.95% lower respectively compared to GPR while its R² value remains 0.1505% higher than GPR.

A thorough evaluation of the seasonal data clearly establishes the GPR model as the leading performer in predicting seasonal GHI with the highest accuracy.

**Table 5:** Drop-one-feature ablation analysis of the GPR model’s performance for Chennai.

Feature removed	RMSE	RMSE Increase	*R*²	*R*² Decrease	Relative impact
Complete Set	0.003	-	0.9999	-	Baseline
CGHI	0.0045	0.0015	0.9994	0.0005	High
SZA	0.0042	0.0012	0.9995	0.0004	High
DNI	0.004	0.001	0.9996	0.0003	Moderate
CDNI	0.0038	0.0008	0.9996	0.0003	Moderate
DHI	0.0037	0.0007	0.9997	0.0002	Moderate
T	0.0035	0.0005	0.9997	0.0002	Low
CDHI	0.0034	0.0004	0.9998	0.0001	Low
RH	0.0033	0.0003	0.9998	0.0001	Low

**Fig. 4:**
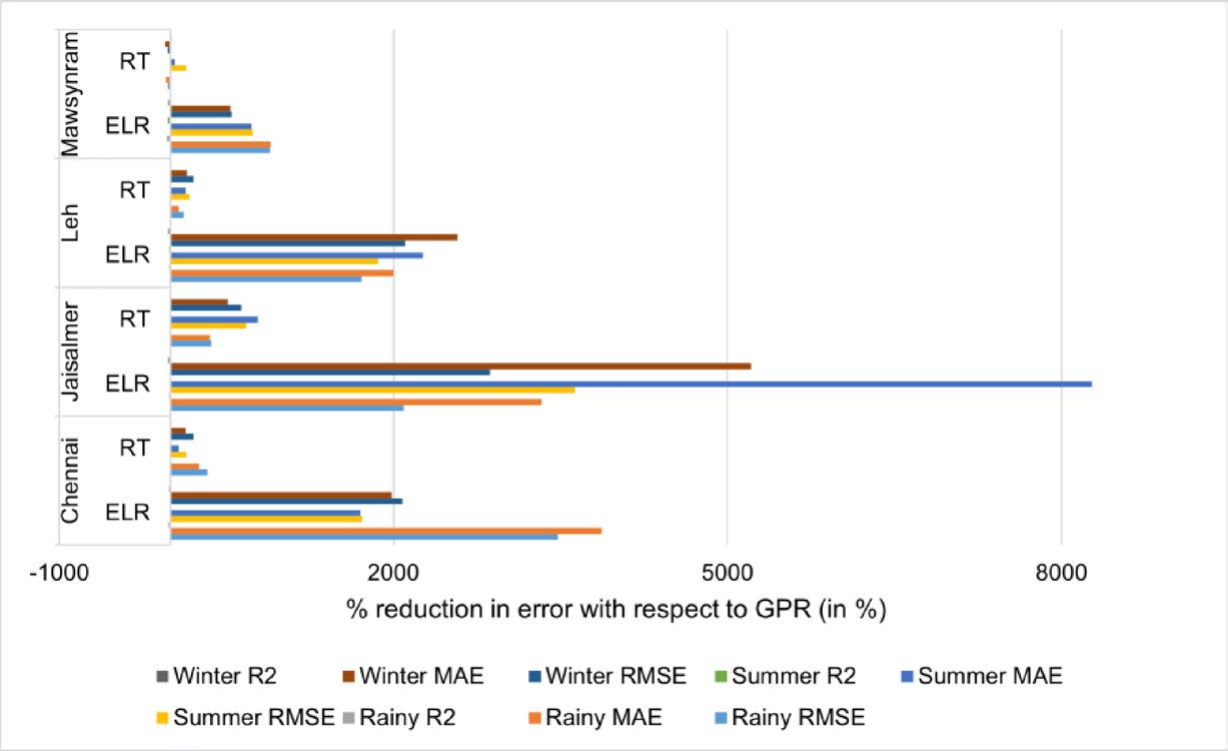
Percentage reduction in error with respect to GPR.

Figure 4 illustrates the percentage reduction in error of the ELR and RT models relative to the GPR model across various seasons and regions. The GPR model serves as the baseline for this comparative analysis. For instance, if we focus on Chennai during the rainy season then RMSE for the ELR and RT models show a significant reduction of 3476.26% and 327.98% respectively when compared to GPR. Similarly, the MAE demonstrates reductions of 3871.18% and 255.96% for ELR and RT respectively against the GPR baseline. Conversely, the R^2^ metric which typically indicates the proportion of variance explained by the model exhibits a reduction of −18.64% for ELR and − 0.25% for RT relative to GPR in this specific scenario. This comprehensive comparison that is conducted across all investigated seasons and regions quantifies the performance improvement measured as a percentage reduction in error achieved by the ELR and RT models when benchmarked against the GPR model.

**Fig. 5:**
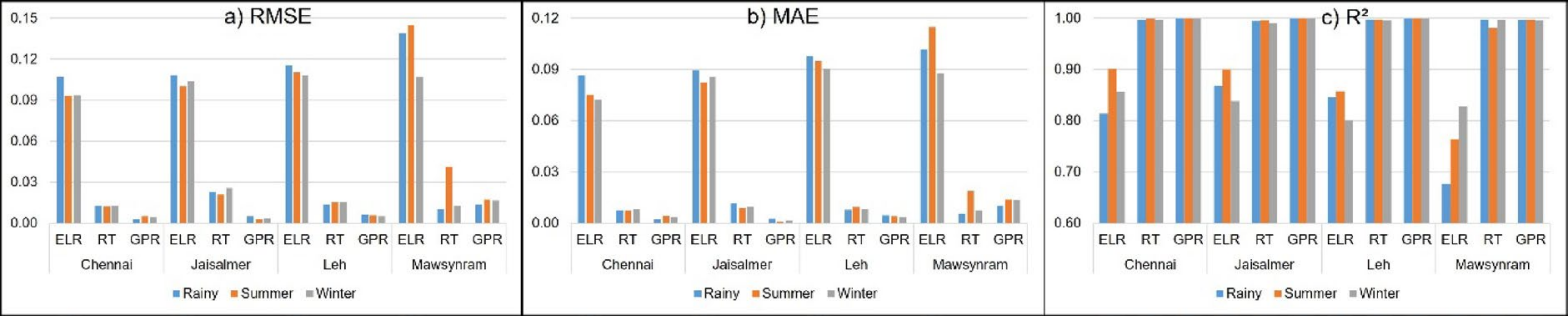
Comparison of seasonal GHI performance using ML models across various Indian locations.

Figure 5. visually compares the performance of three ML models in predicting seasonal GHI across four Indian locations. The chart consists of three subplots. Figure 5a. illustrates the RMSE values for each model, location and season where lower bars indicate better performance with smaller prediction errors. Figure 5b. presents the MAE values with lower bars signifying improved accuracy through smaller average absolute errors. Figure 5c. displays the R² values where higher bars which are closer to 1.00 suggest a better model fit indicating a greater ability to explain the variance in seasonal GHI predictions.

The GPR model consistently ranks as the top-performing approach across all four locations and three seasons based on key evaluation metrics for predicting seasonal GHI. It demonstrates the lowest prediction errors and accounts for the highest proportion of variance in GHI. While the RT model does not match GPR’s performance, it reliably exhibits lower errors and higher R² values compared to ELR. Among the three models, ELR shows the weakest predictive accuracy, displaying the highest RMSE and MAE, as well as the lowest R² values across all locations and seasons.

Although model performance varies slightly depending on location and season, indicating that seasonal GHI predictability is influenced by these factors, the relative ranking of models (GPR > RT > ELR) remains largely consistent. Overall, this analysis strongly suggests that the GPR model is the most accurate and reliable for predicting seasonal GHI across these Indian locations. The RT model performs reasonably well, while the ELR model is comparatively less effective.

**Fig. 6:**
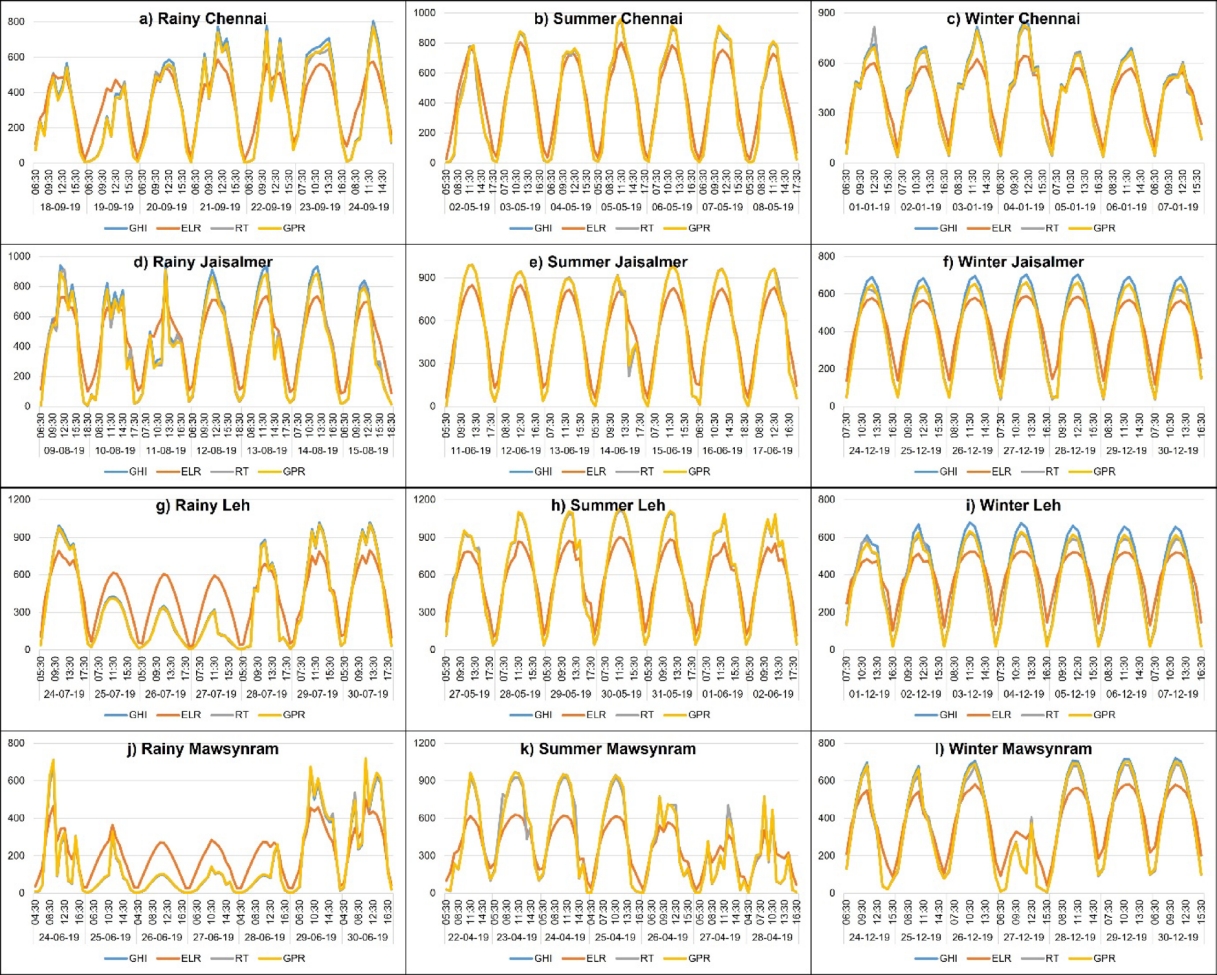
Season-wise depiction of GHI predictions for a randomly selected week across four Indian locations.

Figure 6. provides a season-wise visualization of GHI predictions for a randomly selected week from the dataset across four Indian locations. Seasonal GHI predictions are plotted using three ML models for four seasons alongside actual GHI measurements for comparison. The chart consists of 12 subplots arranged in a 4 × 3 grid where each row corresponds to a specific location and each column represents a season. Within each subplot the x-axis denotes the time of day over the course of a week while the y-axis represents GHI values (in W/m²). Different colored lines distinguish actual GHI observations from predictions made by the three ML models. This structured visualization enables a comparative analysis of model performance across various locations and seasonal conditions. The predictions are examined in detail, providing a comprehensive assessment of model performance across various locations and seasons. Figure 6a, b and c depict Chennai across the rainy, summer, and winter seasons, respectively. Similarly, Fig. 6d and e, and 6f illustrate Jaisalmer’s seasonal variations. Figure 6g and h, and 6i represent Leh during the rainy, summer, and winter seasons, while Fig. 6j and k, and 6l showcase Mawsynram under the same seasonal conditions.

GPR model consistently provides the most accurate visual representation of actual GHI across all four locations and three seasons. Its predictions closely follow the observed trends and fluctuations demonstrating superior alignment surpassing the other models. The RT model ranks second showing reasonable agreement with actual GHI values. In contrast, the ELR model exhibits the most noticeable deviations often underestimating peak values and failing to capture fluctuations as effectively as GPR and RT. The daily cycle of GHI is distinctly visible in most plots, with higher values occurring around midday. As expected, the magnitude of GHI varies significantly depending on location and season. Higher values are observed in summer and in regions with clearer skies such as Jaisalmer and Leh, whereas lower values are recorded during winter and the rainy season particularly in Mawsynram.

#### Drop-one-feature ablation analysis

An ablation analysis is conducted to further validate the robustness of the selected feature set for the Chennai dataset using the best-performing GPR model. The analysis presented in Table 6 shows the performance degradation when each feature is individually removed from the complete eight-feature set. The results demonstrate that each selected feature contributes meaningfully to prediction accuracy. CGHI shows the highest impact as its removal causes an RMSE increase of 0.0015. SZA follows with an RMSE increase of 0.0012 and DNI with an RMSE increase of 0.0010. Even the feature with the smallest individual impact, which is RH, causes measurable performance degradation, leading to an RMSE increase of 0.0003. This confirms that all eight features provide non-redundant predictive information.

**Table 6:** Comparison of seasonal GHI performance using ML models across various Indian locations.

City	Model	Rainy			Summer				Winter			
		RMSE	MAE	R^2^	RMSE		MAE	R^2^	RMSE		MAE	R^2^
Chennai	ELR	0.1070	0.0864	0.8135	0.0932		0.0751	0.9005		0.0933	0.0724	0.8574
	RT	0.0128	0.0077	0.9973	0.0124		0.0073	0.9983		0.0130	0.0081	0.9972
	GPR	**0.0030**	**0.0022**	**0.9999**	**0.0051**		**0.0042**	**0.9997**		**0.0043**	**0.0035**	**0.9997**
Jaisalmer	ELR	0.1083	0.0895	0.8686	0.1000		0.0824	0.8999		0.1038	0.0855	0.8383
	RT	0.0230	0.0118	0.9941	0.0210		0.0087	0.9956		0.0258	0.0098	0.9900
	GPR	**0.0049**	**0.0026**	**0.9997**	**0.0027**		**0.0010**	**0.9999**		**0.0035**	**0.0016**	**0.9998**
Leh	ELR	0.1157	0.0980	0.8455	0.1104		0.0949	0.8580		0.1081	0.0906	0.8005
	RT	0.0140	0.0080	0.9977	0.0153		0.0094	0.9973		0.0151	0.0083	0.9961
	GPR	**0.0064**	**0.0047**	**0.9995**	**0.0056**		**0.0040**	**0.9996**		**0.0049**	**0.0034**	**0.9996**
Mawsynram	ELR	0.1390	0.1018	0.6776	0.1451		0.1147	0.7639		0.1068	0.0877	0.8277
	RT	**0.0104**	**0.0059**	**0.9982**	0.0413		0.0188	0.9808		**0.0130**	**0.0073**	**0.9974**
	GPR	0.0140	0.0102	0.9967	**0.0173**		**0.0139**	**0.9966**		0.0165	0.0137	0.9959

### Feature interaction analysis

Beyond individual feature importance, the interaction patterns among selected features provide insights into their collective predictive behaviour. Feature group analysis reveals complementary information contributions across different meteorological parameter categories. The five irradiance-related features which are CGHI, CDHI, CDNI, DHI and DNI collectively account for 94.2% of the total model variance leading to R² = 0.9420 when used alone, while the three atmospheric features which are T, RH and SZA contribute the remaining predictive information necessary to achieve the full model performance of R² = 0.9999.

Analysis of feature subgroups demonstrates synergistic effects between clearsky and measured irradiance parameters. Clearsky features namely CGHI, CDHI and CDNI alone achieve R² = 0.9654, while measured irradiance features namely DHI and DNI alone achieve R² = 0.9423. However, their combination yields R² = 0.9987, indicating that clearsky conditions provide theoretical upper bounds while measured values capture real-world atmospheric effects, creating complementary predictive information essential for accurate GHI forecasting.

The inclusion of atmospheric parameters such as T and RH alongside solar geometry namely SZA addresses the remaining model variance by capturing local weather conditions that influence the relationship between theoretical and actual irradiance. This multi-dimensional feature representation ensures robust seasonal GHI prediction across diverse climatic conditions.

Our dual-criterion approach using both Spearman correlation for monotonic relationships and MI for non-linear dependencies provides complementary perspectives on feature relevance that capture both linear and complex non-linear relationships with GHI. The threshold-based selection ensures systematic feature inclusion while the hypothesis testing framework provides statistical rigor. This approach is particularly appropriate for meteorological data where relationships may exhibit both monotonic trends and complex interactions.

### Diagnostic validation of GPR model

The GPR model achieves near-perfect R² values of ~ 0.9999 across all seasons and locations indicating a highly accurate fit. However, such elevated performance metrics potentially suggest overfitting or data leakage. To confirm the generalization capability of the model, diagnostic validations are conducted through residual analysis and learning curve assessment.

**Fig. 7:**
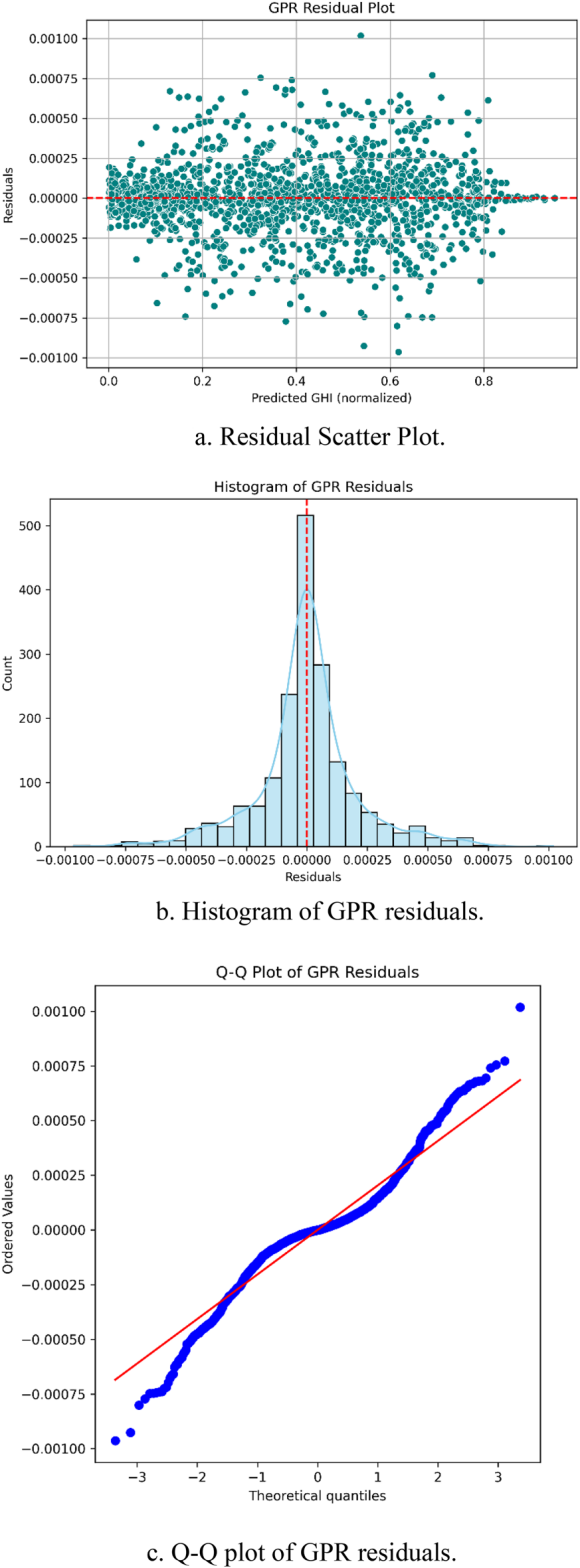
(**a**). Residual Scatter Plot. (**b**). Histogram of GPR residuals. (**c**). Q-Q plot of GPR residuals.

Residual plots are generated by calculating the difference between actual and predicted GHI values. An ideal model exhibits residuals that are randomly distributed around zero with no visible structure. As illustrated in Fig. 7a the residuals of the GPR model are symmetrically dispersed around the zero-line showing no observable patterns. The histogram of residuals shown in Fig. 7b confirms a unimodal and approximately normal distribution while the Q–Q plot in Fig. 7c indicates that the residuals follow a near-normal trend. These results demonstrate that the prediction errors are unbiased and homoscedastic.

**Fig. 8:**
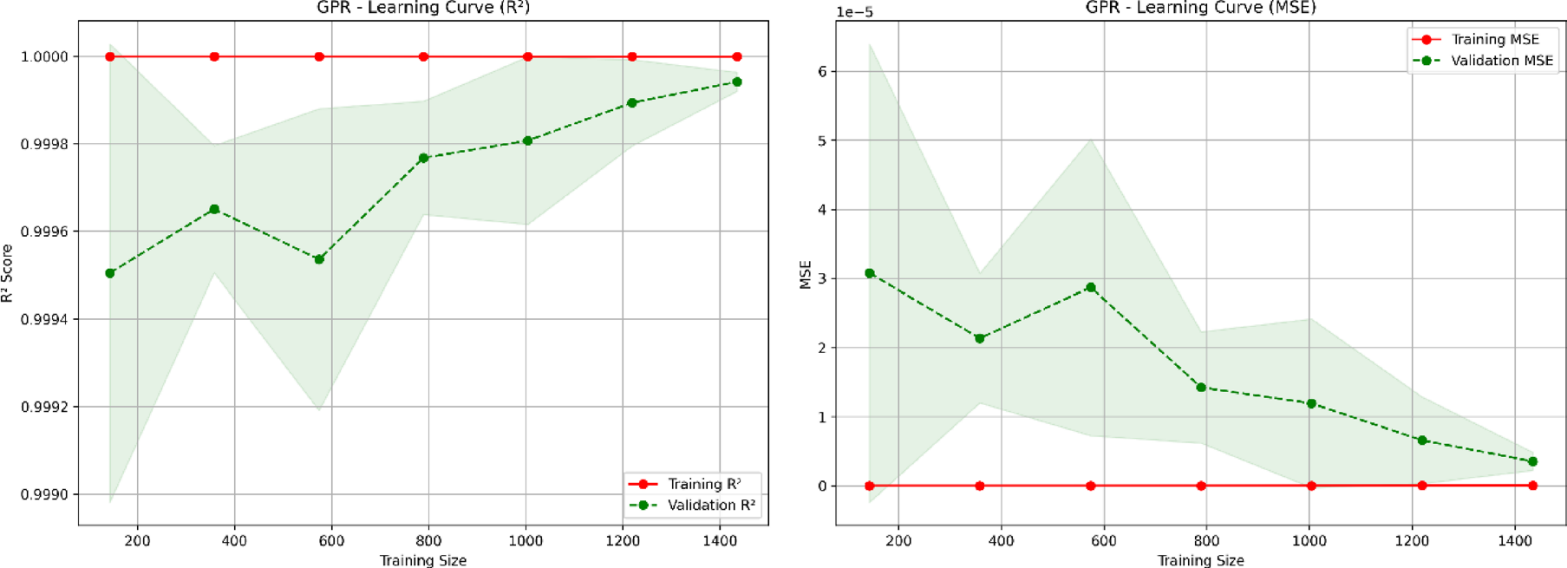
GPR learning curves showing R² on the left and MSE on the right.

To further evaluate the model robustness, learning curves are plotted using both R² and Mean Squared Error (MSE) metrics across increasing training sizes. As shown in Fig. 8 the training and validation scores converge steadily with minimal deviation suggesting that the model does not overfit and learns effectively from limited as well as large datasets. The small and stable generalization gap across training sizes validates the consistency and scalability of the GPR model.

These diagnostic analysis confirms that the high performance of the GPR model stems from its inherent ability to capture complex and nonlinear patterns in meteorological data rather than from overfitting or information leakage. Hence, the model is suitable for reliable seasonal GHI forecasting in diverse climatic regions.

## Conclusion & future scope

Successful management and operation of SAPV systems depend on accurate seasonal GHI forecasting ensuring reliable and cost-efficient energy generation. This study analyses a dataset that comprises of four climatically distinct Indian locations (Chennai, Jaisalmer, Leh and Mawsynram) sourced from the NSRDB data viewer. A comprehensive data quality assessment is conducted addressing missing values and outliers. Significant features relevant to seasonal GHI prediction are identified using Spearman’s correlation coefficient followed by hypothesis testing to determine their statistical significance. During the feature selection process, variables exhibiting weak or no correlation as well as those with low or negligible MI are systematically excluded. Ultimately, eight key features are selected that contribute the most effectively to robust seasonal GHI forecasting. The selected features are then utilized for training and testing three ML models namely ELR, RT and GPR. Model performance is evaluated using error metrics. The results show that GPR consistently outperformed ELR and RT in seasonal GHI predictions across diverse Indian locations. GPR achieves the lowest RMSE of 0.0030 surpassing ELR by 189.1% and RT by 124.05%. It also records the smallest MAE of 0.0022 with 190.09% and 111.1% fewer errors than ELR and RT respectively. Additionally, GPR’s R² score of 0.9999 exceeds ELR and RT models by 20.56% and 0.2604% respectively. While RT provides reasonably accurate forecasts, ELR demonstrates the least reliability.

While this study demonstrates robust temporal generalization across climatically diverse Indian regions, spatial generalization to completely unseen locations represent an important avenue for future research. Cross-site validation would require additional methodological considerations including domain adaptation techniques to address potential distribution shifts in meteorological patterns between geographically distant locations. Transfer learning approaches could enhance model generalizability by leveraging learned patterns from training locations to improve predictions in new geographic regions with limited historical data. Further research directions include expanding the ablation study to investigate higher-order feature interactions, incorporating additional meteorological parameters such as cloud cover indices or aerosol optical depth and developing ensemble methods that combine location-specific models for improved regional forecasting accuracy.

The future scope of seasonal GHI prediction using ML models encompasses several promising research directions and applications: enhancing model accuracy, integrating advanced meteorological features, expanding generalization across diverse geographic regions, incorporating renewable energy planning, developing real-time forecasting systems, improving model explainability and interpretability along with advancing sustainability and climate change adaptation. These innovations will contribute to refining seasonal GHI predictions ensuring more precise and efficient solar energy utilization.

## Data Availability

For this research, we utilized the ‘Europe, Africa & Asia (15, 30, 60 min/4 km/2017–2019)’ dataset, which is available through the NSRDB repository at https://nsrdb.nrel.gov/data-viewer.To ensure transparency and reproducibility, the complete codebase supporting this work which includes the data preprocessing, min-max normalization, feature selection, model training, hyperparameter optimization and evaluation is made publicly available at https://github.com/aadyashapatel2019phd/ghi-forecasting.
